# Supersaturated Oxygen Emulsion Mitigates Hypoxia-Driven Corneal Neovascularization after Alkali Burn

**DOI:** 10.1016/j.xops.2025.100898

**Published:** 2025-07-28

**Authors:** Asmaa A. Zidan, Elsayed Elbasiony, Zhirong Lin, Sheyda Najafi, Kathryn Pate, Jia Yin

**Affiliations:** 1 Department of Ophthalmology, Schepens Eye Research Institute of Mass Eye and Ear, Harvard Medical School, Boston, Massachusetts; 2 Coruna Medical, LLC, Longmont, Colorado

**Keywords:** Alkali burn, Corneal neovascularization, HIF, Hypoxia-inducible factor, Tissue hypoxia

## Abstract

**Purpose:**

Alkali burn is a vision-threatening ocular emergency with no targeted acute therapy. We previously identified tissue hypoxia as a key driver and developed a perfluorodecalin-based supersaturated oxygen emulsion (SSOE) that delivers high levels of oxygen topically. In this study, we aim to investigate the role of hypoxia-inducible factor signaling in postburn sequalae and evaluate the therapeutic efficacy and timing of SSOE in treating ocular alkali burn.

**Design:**

Experimental animal study.

**Subjects:**

A total of 207 BALB/c mice were used in this study. Subjects were assigned to 6 experimental groups: naïve (n = 31), untreated or vehicle-treated controls (n = 75), and treatment groups receiving SSOE either immediately postinjury (n = 33), or with delayed initiation at 1 day (n = 26), 2 days (n = 21), or 5 days (n = 21) after alkali burn. Where possible, mice were used for multiple outcome assessments to reduce total animal use in accordance with ethical and institutional guidelines.

**Methods:**

Alkali burn was induced by applying 1M sodium hydroxide solution to the central cornea of BALB/c mice, followed by immediate or delayed (by 1, 2, or 5 days) topical application of SSOE or vehicle control daily for 14 days. Corneal opacity, neovascularization (NV), and cataract formation were assessed, and hypoxia-inducible factor 1-alpha (HIF-1α) and VEGF expression were measured.

**Main Outcome Measures:**

Corneal NV, anterior chamber (AC) inflammation, cataract formation, and HIF-1α or VEGF expression.

**Results:**

Alkali burn led to persistent HIF-1α activation in the cornea up to day 14 postinjury, which was strongly correlated with corneal NV. Immediate SSOE treatment significantly reduced corneal NV, edema, AC inflammation, cataract formation, and expression of HIF-1α and VEGF at days 14 and 28. Delayed SSOE application (up to 5 days postinjury) also improved corneal NV, edema, inflammation, and fibrosis but did not prevent cataract formation.

**Conclusions:**

Daily SSOE treatment mitigates hypoxia-driven corneal NV by inhibiting HIF-1α and VEGF signaling. Early administration offers the greatest benefit, though delayed treatment remains effective in reducing corneal damage. These findings support the potential of SSOE as a novel topical therapy for chemical eye injuries.

**Financial Disclosure(s):**

Proprietary or commercial disclosure may be found in the Footnotes and Disclosures at the end of this article.

Chemical burns to the eye, particularly by alkaline agents, represent one of the most severe and sight-threatening ocular emergencies, often resulting in irreversible vision impairment and blindness.^1^ Large epidemiological studies in the United States and United Kingdom estimate an incidence of 51 to 56 cases per million annually, with financial burdens reaching millions in emergency department costs.^2^, ^3^, ^4^ These injuries are frequently encountered in industrial settings, household accidents, and military environments, where timely medical intervention may be limited.^5^^,^^6^ Alkali agents rapidly penetrate ocular tissues due to their lipophilic nature, causing saponification of cell membranes, collagen denaturation, and stromal damage. These effects trigger a cascade of inflammation, hypoxia, and angiogenesis, leading to corneal opacification, neovascularization (NV), and fibrosis, as well as intraocular inflammation, cataract formation, and even damage to the retina and optic nerve.^7^^,^^8^ Current therapeutic strategies are limited to prompt irrigation to remove chemicals and inflammation control, using topical corticosteroids and anti-inflammatory agents.^9^ Despite these interventions, permanent damage and vision loss still occur, necessitating further development of more effective treatments.

Our previous study has highlighted the role of hypoxia in acute ocular alkali burn.^7^ Hypoxia stabilizes hypoxia-inducible factor 1-alpha (HIF-1α), driving pathological processes such as inflammation, angiogenesis, and fibrosis.^10^ We have developed a perfluorodecalin (25%)-based supersaturated oxygen emulsion (SSOE) to deliver high levels of dissolved oxygen directly to the injured tissues.^7^ In our previous report, we have shown that a single application of SSOE immediately after an alkali burn reverses ocular hypoxia, dampens acute HIF activation and inflammation, and reduces ocular damage. This single application, however, did not mitigate HIF activation beyond the acute stage or reduce corneal NV and edema.^7^ Corneal NV is not only a primary target of HIF activation but the most important negative prognosticator for corneal transplantation, the ultimate “cure” for corneal blindness.^11^ Thus, further investigation into the duration of HIF activation and the optimal therapeutic regimen for targeting hypoxia and the HIF pathway to mitigate alkali-induced ocular damage is warranted.

In this study, we first characterized the pattern of hypoxia and HIF activation up to 28 days after alkali burn. We then investigated the efficacy of prolonged daily SSOE treatment in reducing persistent HIF activation and mitigating corneal NV after burn. Lastly, we examined the therapeutic window of SSOE treatment with delayed application up to 5 days after burn. These findings provide critical insights into the mechanism of action and feasibility of SSOE as a topical treatment for chemical burns, addressing both immediate and delayed intervention scenarios.

## Methods

### Animal

This study was conducted following the Association for Research in Vision and Ophthalmology Statement for the Use of Animals in Ophthalmic and Vision Research and approved by the institutional animal care and use committee. Adult males and females BALB/c mice aged 8 to 10 weeks were used. The mice were housed in pathogen-free conditions under controlled temperature and humidity, with a 12-hour light–dark cycle. While we did not enforce strict sex matching in each individual experiment due to practical constraints and availability, we ensured a roughly equal distribution of sexes across the full set of experiments.

### Ocular Alkali Burn Model

Corneal alkali burn was induced as previously described.^7^ Briefly, a sterile 2-mm filter paper disc saturated with 1M sodium hydroxide was carefully applied to the central cornea, sparing the limbus, for 20 seconds. Excess sodium hydroxide was blotted off with sterile paper, and the ocular surface was thoroughly irrigated with saline for 5 minutes until pH levels returned to neutral. After the injury, triple antibiotic eye ointment was applied 3 times daily for the first 3 days to prevent infection. This regimen was applied consistently across all experimental groups.

## Measurement of Anterior Chamber Oxygen Concentration

Oxygen levels in the anterior chamber (AC) were measured using the DP-PSt7-2 oxygen sensor (PreSens). After anesthesia induction, a 28-gauge needle was used to create a narrow self-sealing tunnel in the temporal cornea near the limbus. To ensure precise sensor placement, the needle tip was premarked with surgical dye (Accu-line Products Inc) before insertion into the AC. Oxygen was measured on days 7 and 14 postinjury.

### Topical SSOE Treatment

Supersaturated oxygen emulsion was formulated as previously described.^7^ Briefly, 25% (w/v) perfluorodecalin was homogenized with Phospholipon 90H, Polawax, and water; the emulsified nanoparticles were then supersaturated with medical-grade oxygen gas in a high-pressure reaction vessel. The final SSOE was packaged in small pressurized dispensing canisters. For topical application, 50 μL of SSOE emulsion was applied either immediately or delayed (by 1 or 2 days) after injury to the ocular surface for 30 minutes while the mice remained anesthetized, followed by once daily 5-minute treatment as a topical ophthalmic emulsion for 14 days. Another group of mice received SSOE treatment 5 days after burn for a total of 14 days with a 4-times-daily regimen to enhance therapeutic effects. An unoxygenated emulsion containing the same ingredient as SSOE was used as vehicle control. Our previous report found no toxicity of SSOE or the vehicle emulsion and the vehicle-treated eyes had a similar presentation to the burned and untreated eyes.^7^

### Clinical and Anterior Segment OCT Evaluation

Corneal opacity, NV, and cataract formation were evaluated using slit lamp biomicroscopy (Topcon). Corneal NV was graded using a scoring system (0–12) based on vessel length and density, where 0 indicated no vessels and 3 represented complete vascularization in each of the 4 quadrants.^12^ Anterior segment OCT imaging (Bioptigen Spectral Domain Ophthalmic Imaging System, Envisu R2200) was performed to assess anterior segment structural changes. Measurements included central corneal thickness (CCT) and AC depth at days 7, 14, 21, and 28 postinjuries.

### Flow Cytometry Analysis

Corneal tissues were harvested on days 7, 14, and 28 and digested enzymatically into single-cell suspensions. Cells were stained with antibodies targeting HIF-1α-APC (Bio legend) and VEGF-fluorescein isothiocyanate (Bio legend). Flow cytometry was performed to quantify marker expression levels, with data presented as percentages of positive cells. Gating strategies were based on previously established protocols to ensure reproducibility.

### Real-Time Polymerase Chain Reaction

Total RNA was extracted from mouse corneal tissues using the RNeasy Micro kit (Qiagen), and complementary DNA synthesis was performed with the SuperScript III kit (Thermo Fisher Scientific Inc) following the manufacturer's instructions. Quantitative real-time polymerase chain reaction was conducted on the LightCycler 480 II System (Roche Applied Science GmbH). All experiments were conducted in duplicate, with data normalized to the housekeeping gene glyceraldehyde 3-phosphate dehydrogenase mRNA levels.

### Histology and Immunohistochemistry

The eyes were harvested from mice, fixed in formalin, and embedded in paraffin, then sectioned and stained with hematoxylin and eosin for examination under bright-field microscopy. For immunohistochemistry, the sections were deparaffinized and rehydrated, followed by enzymatic antigen retrieval using pepsin (Sigma Aldrich). Sections were then washed and blocked with 2% bovine serum albumin and 0.1% Triton X-100 for 1 hour at room temperature. The slides were then incubated overnight at 4°C with CD45 or alpha smooth muscle actin antibodies (Abcam). After washing with phosphate buffered saline with Tween 20, the slides were incubated with appropriate fluorescently labeled secondary antibodies diluted in 1% bovine serum albumin, washed 3 times, and mounted with Vectashield containing 4′,6-diamidino-2-phenylindole. Corneal NV was assessed using corneal whole mounts. Briefly, corneal tissue was collected, fixed in 4.5% paraformaldehyde, permeabilized with Triton X, and stained overnight with CD31 or Lyve-1. The following day, the corresponding secondary antibody was added. After several washes, the tissues were mounted. All the slides were visualized using a confocal laser scanning microscope (SP8, Leica). Quantitative analysis using ImageJ (National Institutes of Health) was performed to measure marker expression.

### Statistical Analysis

Statistical analyses were conducted using GraphPad Prism software (version 10). All data are presented as mean ± standard error of the mean. One-way analysis of variance with Tukey post hoc tests was applied for multiple comparisons, and an unpaired *t* test was applied to the 2 groups' comparison. Fisher exact test was used to compare cataract frequency between groups. Pearson correlation coefficient was calculated to assess the relationship between NV and HIF frequency. *P* values <0.05 were considered statistically significant. We did not observe any notable sex-related differences in response across the parameters assessed. Therefore, data from both sexes were pooled for analysis.

## Results

### Persistent Ocular Hypoxia and HIF Activation After Alkali Burn

**Figure 1:**
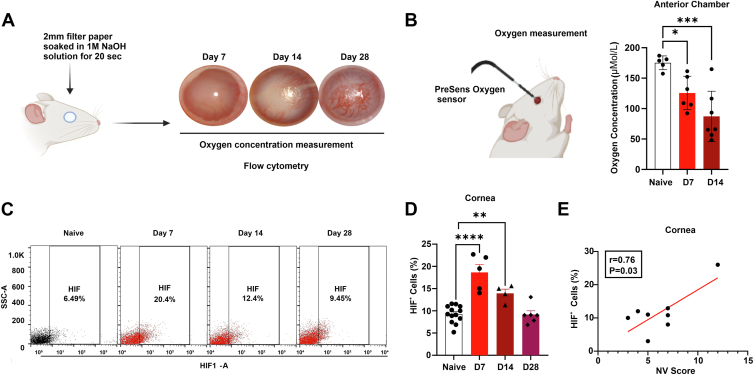
Persistent ocular hypoxia and HIF activation after alkali burn. **A,** Schematic showing the injury model and the timeline for tissue collection postinjury. **B,** Oxygen concentration in the anterior chamber was recorded in vivo with a micro-oxygen sensor and showed significant decrease on day 7 and day 14 after alkali burn (n = 5 for naïve group, n = 6 for day 7 group, n = 7 for day 14 group). **C,** Representative flow cytometry plot illustrating the frequencies of HIF^+^ cells in the corneas at days 7, 14, and 28 postburn. **D,** Flow cytometry analysis of the cornea shows that the frequency of HIF^+^ cells was significantly higher at days 7 and 14 postburn compared with naïve controls (n = 13 for naïve group, n = 5 for day 7 group, n = 4 for day 14 group, n = 6 for day 28 group). **E,** The corneal NV score showed a strong positive correlation with the percentage of HIF^+^ cells in the cornea at day 14 (n = 8). Data are presented as mean ± SEM. Statistical significance was determined using 1-way ANOVA (**B**, **D**) and Pearson correlation (**E**), ∗*P* < 0.05, ∗∗*P* < 0.01, ∗∗∗*P* < 0.001, ∗∗∗∗*P* < 0.0001. Figure A & B were created with BioRender.com. ANOVA = analysis of variance; HIF = hypoxia-inducible factor; NaOH = sodium hydroxide; NV = neovascularization; SEM = standard error of the mean; SSC-A = side scatter area.

Ocular chemical burn was induced by applying a 2-mm filter paper soaked in 1 M sodium hydroxide to the central cornea for 20 seconds, followed by thorough irrigation with saline for 5 minutes. This well-established injury model resulted in corneal opacity, cataract formation, and progressive NV, as observed with slit lamp imaging (Fig 1A). Using this model, we previously demonstrated a rapid onset of tissue hypoxia, evidenced by a decrease in intraocular oxygen concentration from 165.4 ± 20.3 μmol/L to 66.1 ± 15.6 μmol/L within seconds of injury, accompanied by acute HIF activation in 33.5 ± 2.6% of total corneal cells at day 1 postburn.^7^ Here we investigated the role of subacute and chronic hypoxia and HIF activation.

We measured the oxygen concentration in the AC and found a significant decrease at day 7 (125.5 ± 11.2 μMol/L, *P* = 0.03) and day 14 (87.2 ± 15.7 μMol/L, *P* = 0.0004), compared with naïve (175.5 ± 5 μMol/L) (Fig 1B). Flow cytometry analysis of corneal tissues revealed that alkali burn induced a significant increase in HIF^+^ cell frequencies at days 7 (18.6 ± 1.8%, *P* < 0.0001) and 14 (13.9 ± 0.9%, *P* = 0.008) compared with naïve controls (9.3 ± 0.5%). By day 28, HIF^+^ cell frequencies returned to baseline levels (9.1 ± 0.9%, *P* = 0.9) (Fig 1C, D). Moreover, we found a strong positive correlation (R = 0.76, *P* = 0.03) between the extent of corneal NV (clinical scores with masked grading) and the percentage of HIF^+^ cells in the cornea on day 14 (Fig 1E). These data suggest that tissue hypoxia and HIF activation persist beyond the acute stage of ocular burn and are likely the drivers for progressive corneal NV.

### Daily SSOE Application Suppresses Corneal NV, Edema, Cataract Formation, and Intraocular Inflammation

**Figure 2:**
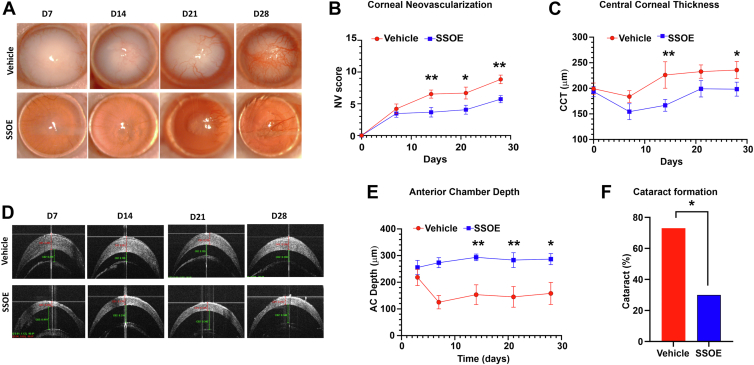
Daily SSOE application suppresses corneal NV, edema, cataract formation, and intraocular inflammation after alkali burn. **A,** Representative slit lamp photographs of vehicle- and SSOE-treated eyes up to 28 days postinjury. Vehicle-treated controls displayed progressive corneal NV and optical opacity, while SSOE-treated eyes exhibited reduced NV and opacity. **B,** Corneal NV scores were significantly lower in the SSOE-treated group (n = 10 per group). **C,** Supersaturated oxygen emulsion treatment significantly reduced CCT compared with vehicle-treated controls at days 14 and 28 (n = 10 per group). **D,** Representative AS-OCT images demonstrated a significant decrease in CCT and an increase in AC depth in SSOE-treated mice compared with vehicle-treated controls. **E,** Supersaturated oxygen emulsion treatment significantly increased AC depth compared with vehicle-treated controls on days 14, 21, and 28 (n = 10 per group). **F,** Supersaturated oxygen emulsion treatment significantly reduced cataract formation compared with vehicle-treated controls on day 28 (n = 15 per vehicle, n = 10 per SSOE). Data are presented as mean ± SEM. Statistical significance was determined by unpaired *t* test (**B**, **C**, **E**) and Fisher exact test (**F**), ∗*P* < 0.05, ∗∗*P* < 0.01. AC = anterior chamber; AS-OCT = anterior segment OCT; CCT = central corneal thickness; NV = neovascularization; SEM = standard error of the mean; SSOE = supersaturated oxygen emulsion.

In our previous study,^7^ we demonstrated that a single 30-minute topical application of SSOE significantly increased oxygen levels in the AC, with elevated oxygen concentrations sustained for up to 150 minutes postapplication. A single immediate application of SSOE was able to treat alkali burn and proved to be effective in reducing corneal fibrosis, optical opacity, cataract formation, and intraocular inflammation, but not effective against corneal NV or edema.^7^ In the current study, SSOE was applied topically as an ophthalmic emulsion immediately after injury and continued once daily for 14 days. Supersaturated oxygen emulsion treatment significantly reduced optical opacity and corneal NV compared with vehicle-treated controls (Fig 2A). Daily SSOE-treated corneas exhibited significantly lower NV scores on days 14 (3.7 ± 0.8, *P* = 0.007) and 28 (5.7 ± 0.7, *P* = 0.002), compared with vehicle-treated corneas (day 14: 6.5 ± 0.6; day 28: 8.8 ± 0.7) (Fig 2B). Additionally, daily SSOE-treated corneas had less corneal edema, evidenced by the significantly lower CCT measured with anterior segment OCT at day 14 (166.7 ± 11.6 μm vs. 226.2 ± 25.9 μm, *P* = 0.009) and day 28 (198.4 ± 13.7 μm vs. 235.7 ± 16.7 μm, *P* = 0.04), compared with vehicle controls (Fig 2D, E). Consistent with our prior report with single application,^7^ daily SSOE treatment prevented intraocular inflammation and exudation, as evidenced by the deeper AC depth in SSOE-treated eyes on day 14 (293.6 ± 12.4 μm vs. 153.6 ± 30.7 μm, *P* = 0.002) and day 28 (286.9 ± 20.6 μm vs. 158.4 ± 41.3 μm, *P* = 0.01) compared with the vehicle group (Fig 2E). Cataract formation was markedly reduced, with only 30% (n = 10) in the SSOE group, compared with 73.3% (n = 15) in the vehicle control group (Fig 2F, *P* = 0.04). These data demonstrate that prolonged SSOE treatment for 14 days offers additional therapeutic benefits, including reducing corneal NV and edema, over the 1-time–only immediate postburn treatment, while maintaining the efficacy in reducing overall optical opacity, cataract formation, and intraocular inflammation.

### Daily SSOE Application Decreases Alkali Burn–Induced Subacute HIF Activation, VEGF Expression, and Angiogenesis

**Figure 3:**
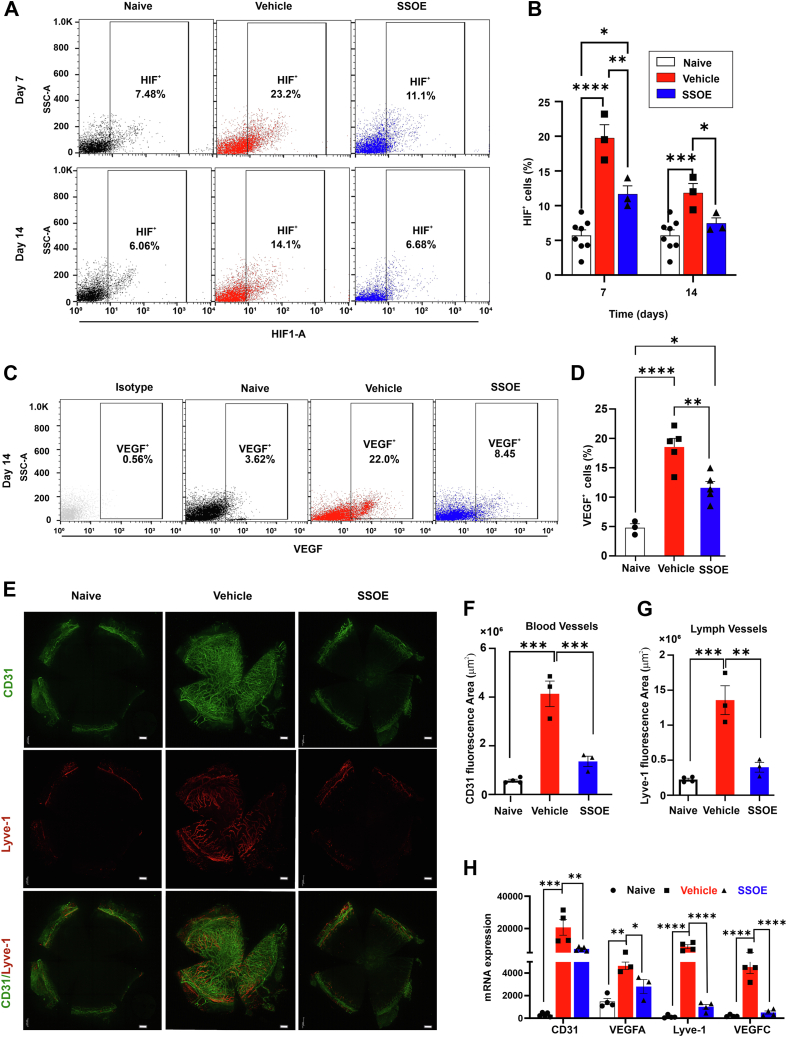
Daily SSOE application decreases alkali burn–induced subacute HIF activation, VEGF expression, and angiogenesis. **A,** Representative flow cytometry plots showing that SSOE treatment suppressed HIF expression on days 7 and 14 postinjury compared with vehicle-treated controls. **B,** The frequency of HIF^+^ cells was significantly lower in corneas from SSOE-treated mice compared with controls, (n = 10 per naïve group, n = 3 per other groups). **C,** Representative flow cytometry plots showing that SSOE treatment suppressed VEGF expression on day 14 postinjury compared with vehicle-treated controls. **D,** The frequency of VEGF^+^ cells was significantly lower in corneas from SSOE-treated mice compared with controls, (n = 3 per naïve group, n = 5 per vehicle group, n = 4 per SSOE group). **E,** Corneas from SSOE-treated mice collected on day 28 exhibited fewer blood (green) and lymph (red) vessels compared with vehicle-treated corneas (scale bar = 300 μm). Immunohistochemical analysis revealed significantly reduced blood (**F**) and lymph (**G**) vessel areas in the SSOE-treated group (n = 3 per group). **H,** Real-time PCR analysis of cornea tissues collected from mice on day 14 showed significantly lower expression of the angiogenic markers (VEGF-A, VEGF-C, CD31, Lyve-1) in the SSOE-treated corneas compared with the vehicle-treated corneas (n = 3 per group). Statistical significance was determined using 1-way ANOVA, ∗*P* < 0.05, ∗∗*P* < 0.01, ∗∗∗*P* < 0.001, ∗∗∗∗*P* < 0.0001. ANOVA = analysis of variance; HIF = hypoxia-inducible factor; PCR = polymerase chain reaction; SSOE = supersaturated oxygen emulsion; SSC-A = side scatter area.

We previously showed that a single application of SSOE immediately after burn significantly reduced HIF-1α activation on day 1 after alkali burn, but had little effect on day 7.^7^ Here, we evaluated the effect of daily SSOE on subacute HIF signaling. Flow cytometry analysis revealed that HIF^+^ cell frequencies were significantly reduced in SSOE-treated corneas at day 7 (11.7 ± 1.2%, *P* = 0.005) and day 14 (7.5 ± 0.8%, *P* = 0.03), compared with vehicle-treated controls (19.8 ± 1.9% on day 7, and 11.8 ± 1.4% on day 14) (Fig 3A, B). VEGF is a downstream effector of HIF and a key promotor of angiogenesis. We found that SSOE treatment resulted in a significantly lower frequency of VEGF^+^ cells in the cornea at day 14 (11.6 ± 1.1%, *P* = 0.005) compared with controls (18.5 ± 1.5%) (Fig 3C, D).

To further validate these findings, immunohistochemical staining was used to assess blood and lymphatic vessels in the corneas. Supersaturated oxygen emulsion–treated corneas exhibited significantly reduced heme- (CD31, *P* = 0.0009) and lymph- (Lyve-1, *P* = 0.002) angiogenesis compared with controls (Fig 3E–G). Similarly, real-time polymerase chain reaction analysis of cornea tissues collected 14 days after the alkali burn revealed that SSOE-treated corneas exhibited significantly reduced expression of the heme-angiogenic markers (VEGF-A, *P* = 0.04; CD31, *P* = 0.007) and the lymph-angiogenic markers (VEGF-C, *P* < 0.0001; Lyve-1, *P* < 0.0001), compared with the vehicle-treated corneas (Fig 3H). These data demonstrate that daily SSOE application suppresses HIF activation and its downstream VEGF-dependent angiogenic response in the subacute phase after alkali burn.

### Delayed SSOE Treatment Reduces Alkali Burn–Induced Corneal Damage but Not Cataract Formation

**Figure 4:**
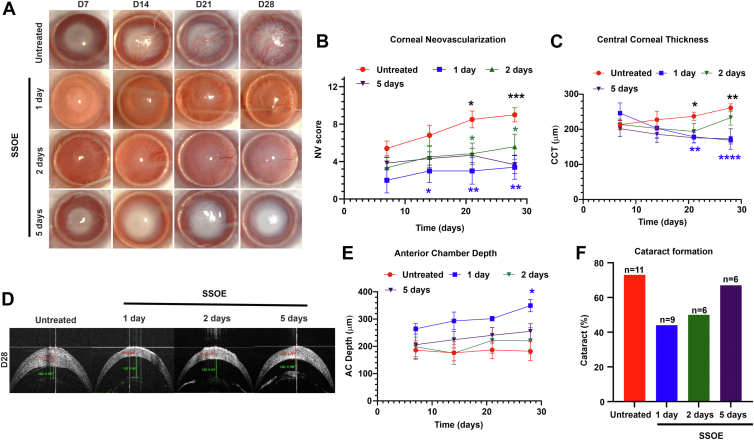
Delayed SSOE treatment inhibits corneal NV but not cataract formation after alkali burn. **A,** Representative slit lamp photographs of untreated and SSOE-treated eyes up to 28 days postinjury. Untreated controls showed progressive corneal NV and optical opacity, whereas SSOE daily treatment (delayed up to 5 days) exhibited reduced NV. **B,** Corneal NV scores were significantly lower in SSOE daily-treated mice, regardless of treatment delay (n = 10 for untreated group; n = 5 for 1-day-delayed group; n = 6 for 2- and 5-day-delayed groups). **C,** Supersaturated oxygen emulsion daily treatment significantly reduced CCT compared with untreated controls at day 28, but only when treatment was delayed by 1 day; no significant difference was observed with 2- or 5-day delays (n = 16 for untreated group; n = 9 for 1-day-delayed group; n = 6 for 2- and 5-day-delayed groups). **D,** Representative AS-OCT images demonstrated significant decreases in CCT and increases in AC depth in SSOE daily-treated mice when treatment was delayed by 1 day, compared with untreated controls. **E,** Daily SSOE treatment significantly increased AC depth compared with untreated controls on day 28, but only with a 1-day treatment delay; no effect was seen with 2- or 5-day delays (n = 16 for untreated group; n = 5 for 1-day-delayed group; n = 6 for 2- and 5-day-delayed groups). **F,** Delayed daily SSOE treatment did not result in a significant effect on cataract formation compared with untreated controls at day 28 delays (n = 11 for untreated group; n = 9 for 1-day-delayed daily treatment group; n = 6 for 2- and 5-day-delayed daily treatment groups). Data are presented as mean ± SEM. Statistical significance was determined using unpaired *t* test (**B**, **C**, **D**), and Fisher exact test (**F**), ∗*P* < 0.05, ∗∗*P* < 0.01, ∗∗∗*P* < 0.001. Blue asterisks indicate 1 day vs. untreated, green asterisks indicate 2 day vs. untreated, black asterisks indicate 5 days vs. untreated. AC = anterior chamber; AS-OCT = anterior segment OCT; CCT = central corneal thickness; NV = neovascularization; SEM = standard error of the mean; SSOE = supersaturated oxygen emulsion.

Chemical burns are common in the battlefield, where immediate treatment may not always be feasible. To determine whether delayed daily SSOE treatment retains therapeutic efficacy, we initiated SSOE administration at 1, 2, or 5 days postinjury. For the 5-day-delayed group, SSOE was applied 4 times per day to enhance its effect. All 3 delayed treatment regimens effectively inhibited corneal NV, but their impact on cataract formation was limited (Fig 4).

**Figure 5:**
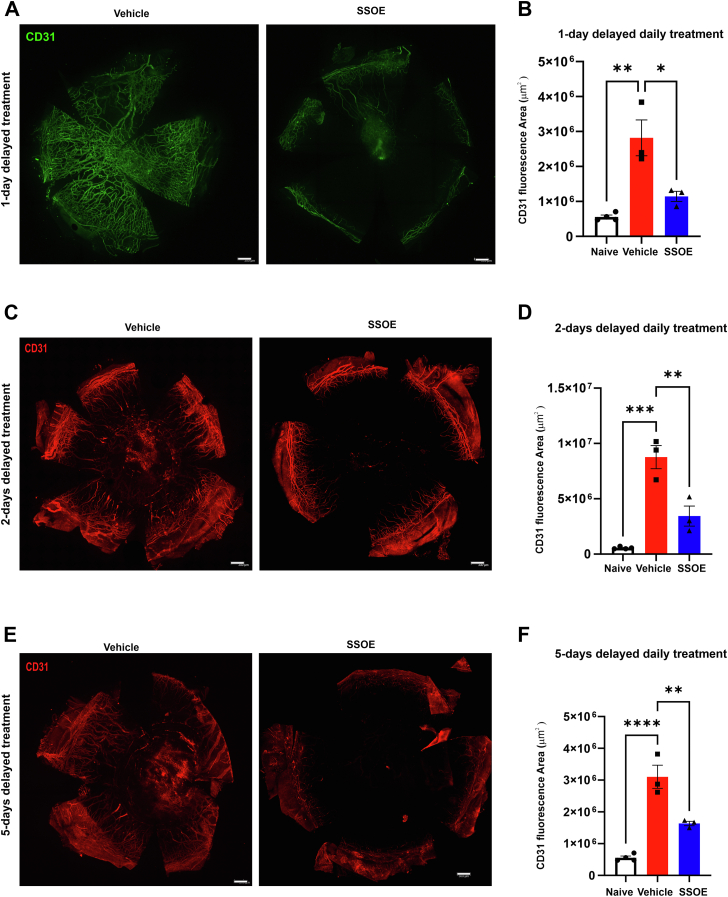
Delayed SSOE treatment reduces corneal neovascularization after alkali burn. **A,** Supersaturated oxygen emulsion–treated corneas 1 day after injury exhibited fewer blood vessels (CD31+ green) compared with vehicle-treated corneas. **B,** Immunohistochemical analysis showed a significantly reduced blood vessel area in SSOE-treated corneas (n = 3 per group). **C, D,** Supersaturated oxygen emulsion–treated corneas, delayed by 2 days from injury, showed a significantly reduced number of blood vessels (CD31+ red) compared with vehicle-treated corneas (n = 3 per group). **E, F,** Supersaturated oxygen emulsion–treated corneas, delayed by 5 days from injury, exhibited significantly fewer blood vessels (CD31^+^ red) compared with vehicle-treated corneas (n = 3 per group). Scale bar = 300 μm. Data are presented as mean ± SEM. Statistical significance was determined using 1-way ANOVA, ∗*P* < 0.05, ∗∗*P* < 0.01, ∗∗∗*P* < 0.001, ∗∗∗∗*P* < 0.0001. ANOVA = analysis of variance; SEM = standard error of the mean; SSOE = supersaturated oxygen emulsion.

Among the delayed daily treatments, the 1-day-delayed group showed the most substantial therapeutic effects, including reductions in NV scores (3.4 ± 1.3 vs. 9 ± 0.8, *P* = 0.002) and significant improvements in CCT (168.9 ± 10.6 μm vs. 261 ± 11.3 μm, *P* < 0.0001) and AC depth (349.5 ± 22.8 μm vs. 182.1 ± 34.5 μm, *P* = 0.02) on day 28, compared with the untreated controls. There was a noted decrease in cataract formation (44% vs. 73%), but it did not reach statistical significance (*P* = 0.2), (Fig 4). The 2-day-delayed daily treatment group also demonstrated reductions in NV scores on day 28 (5.6 ± 1.3, *P* = 0.03) but no significant improvements in cataract formation (50%, n = 6, *P* = 0.4), CCT (233.4 ± 21.5 μm, *P* = 0.2) or AC depth (220 ± 48.3 μm, *P* = 0.5). The 5-day-delayed 4-times-daily treatment group exhibited reductions in NV scores (3.7 ± 0.9, *P* = 0.0007) and CCT (172 ± 29.4 μm, *P* = 0.002) but failed to significantly improve cataract formation (67%, *P* = 0.9) or AC depth (255 ± 27.9 μm, *P* = 0.2) compared with untreated (Fig 4C–F). Regardless of the length of treatment delay, immunohistochemistry performed on day 28 revealed a decrease in the density of CD31 blood vessels in the SSOE-treated corneas compared with untreated corneas (Fig 5).

Anterior chamber inflammation and cataract formation may involve early inflammatory processes, which was unopposed in the delayed SSOE treatment regimens. On the other hand, corneal damages including NV and opacification depend on continuous tissue hypoxia and HIF activation, the inhibition of which by delayed SSOE treatment still effectively reduced angiogenesis. These findings emphasize the importance of timely intervention to optimize therapeutic outcomes.

### SSOE Treatment Reduces Corneal Fibrosis and Inflammation

**Figure 6:**
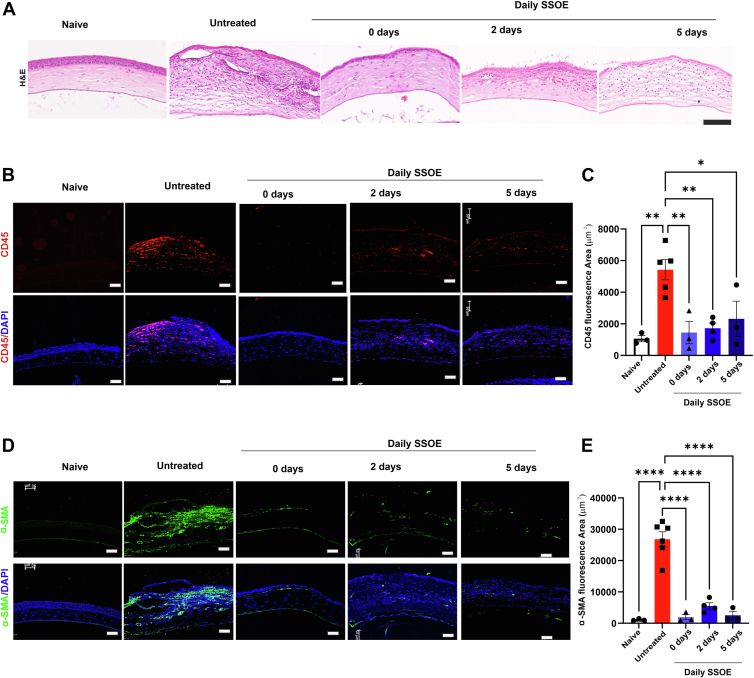
Supersaturated oxygen emulsion treatment reduces corneal fibrosis and inflammation after alkali burn. **A,** Histological analysis using hematoxylin and eosin staining revealed reduced corneal thickness and inflammatory cell infiltration after SSOE treatment, compared with untreated alkali-burned corneas on day 28 (scale bar = 100 μm). **B, C,** Corneas from SSOE-treated mice exhibited significantly lower infiltration of CD45^+^ cells (red) compared with untreated controls (scale bar = 50 μm). **D, E,** Corneas from SSOE-treated mice showed significantly reduced expression of α-SMA (green) compared with untreated controls (scale bar = 50 μm). **C, E,** n = 6 for the untreated group, n = 3 for the SSOE-treated group, data are presented as mean ± SEM. Statistical significance was determined using unpaired *t* test, ∗*P* < 0.05, ∗∗*P* < 0.01, ∗∗∗∗*P* < 0.0001. α-SMA = alpha smooth muscle actin; H&E = hematoxylin and eosin; SEM = standard error of the mean.

Besides NV and edema, alkali burns also induce corneal stromal fibrosis and scar formation.^13^ On day 28, corneal tissues were collected for hematoxylin and eosin staining and showed that alkali burns induced epithelial thinning, stromal edema, NV, and inflammatory infiltration in untreated corneas. In contrast, daily SSOE-treated corneas exhibited reduced thickness and inflammation, particularly when treatment was initiated immediately (Fig 6A). Immunohistochemistry further demonstrated decreased infiltration of CD45^+^ inflammatory cells in daily SSOE-treated corneas regardless of the treatment regimen, compared with the untreated one (Fig 6B, C). Additionally, alpha smooth muscle actin expression, a marker of myofibroblast differentiation and fibrosis, was significantly reduced in all SSOE-treated groups (Fig 6D, E). The effects of daily SSOE treatment on corneal fibrosis and inflammation are similar to those of the 1-time-only treatment we previously reported.^7^

## Discussion

Chemical eye injuries are ophthalmic emergencies with blinding sequelae and limited treatment options. We previously showed that there is acute intraocular tissue hypoxia and HIF activation after alkali burn, and reversing it with an immediate 1-time topical application of SSOE partially mitigates ocular damage.^7^ We expanded this work in the current study and demonstrated that there is persistent tissue hypoxia and HIF activation beyond the acute stage, which strongly correlates with corneal angiogenesis. Additionally, we showed that extended daily application of SSOE suppresses persistent HIF activation and nearly all aspects of alkali burn–induced ocular damages including corneal angiogenesis, edema, opacification, AC inflammation, and cataract formation. Moreover, we showed that delayed application of SSOE for up to 5 days is still effective against corneal NV and fibrosis but has limited efficacy for intraocular inflammation and cataract formation. Collectively, this work demonstrates the critical role of persistent ocular hypoxia in chemical eye injury and the therapeutic potential and treatment window of SSOE in acute and subacute ocular burns.

The cornea is an avascular structure that relies on atmospheric oxygen and aqueous humor for its oxygen supply.^14^ Disruptions in oxygen availability or metabolic demands lead to corneal edema, NV, and loss of transparency.^15^ Persistent hypoxia has been implicated in several corneal diseases, including contact lens-induced NV^16^ and herpes simplex virus keratitis.^17^ Hypoxia stabilizes the HIF-α subunits, which translocate to the nucleus, dimerize with hypoxia-inducible factor beta, and activate the transcription of genes involved in angiogenesis, metabolic, and survival pathways.^18^ Hypoxia-inducible factors also play a crucial role in fibroblast activation and fibrosis by promoting extracellular matrix remodeling, fibroblast proliferation, and collagen stabilization.^19^ Previous studies have confirmed an immediate decrease in oxygen concentration after alkali burns in mice and rabbits,^7^^,^^8^ and we showed that this hypoxia results in HIF activation.^7^ Herein, we demonstrate that, beyond the initial insult, there is a sustained state of ocular tissue hypoxia and HIF activation for up to 14 days postinjury. The persistence of hypoxia after chemical ocular injury likely reflects a complex interplay between tissue damage, vascular disruption, and inflammation. Initially, direct injury to the limbal and iris vasculature reduces oxygen delivery, but the ensuing inflammatory response may further exacerbate hypoxia by increasing metabolic demand and impairing perfusion. In this context, inflammation can be both a cause and a consequence of hypoxia.^20^ This persistent activation coincides with findings from Rao et al,^17^ who reported that in herpes stromal keratitis, a chronic inflammatory condition of the cornea induced by herpes simplex virus-1 infection, there is development of inflammatory hypoxia and increased glycolytic metabolism in progressing lesions. In their study, HIF activation was observed during the clinical disease period, which typically spans from day 6 to day 15 postinfection in the mouse model.^17^ Together, these findings highlight prolonged hypoxia as a common feature in chronic ocular inflammation, supporting hypoxia-targeting therapies.

We found that daily application of SSOE suppresses this continuous HIF activation and its downstream effector, VEGF, and inhibits corneal NV and edema, whereas a single application had no effect.^7^ Notably, SSOE remained effective in reducing corneal NV and stromal fibrosis even when treatment was delayed up to 5 days postinjury, provided it was applied continuously for 14 days. This suggests that ongoing HIF activation is likely necessary to sustain the contiguous growth of neovessels and suppressing it even in the subacute stage of burn is still effective in dampening its downstream effector VEGF and the angiogenic response.

In addition to the daily SSOE regimen for alkali burn, we tested its delayed application for up to 5 days. We noted an interesting dichotomy in the treatment efficacy. While all daily treatment regimens, regardless of initiation time point, effectively mitigated corneal NV, inflammation, and fibrosis, improvements in AC exudates (measured by their depth) were primarily observed with early treatment that was given either immediately or 1 day after the burn. Notably, only immediate treatment significantly reduced cataract formation. Cataract formation after alkali burns is attributed to the rapid penetration of alkali substances through ocular tissues.^21^ These agents saponify cell membrane lipids, facilitating swift entry into the AC, leading to protein denaturation and lens opacification.^22^ Concurrently, an acute inflammatory cascade triggers neutrophil infiltration, proteolytic enzyme release, and oxidative stress, leading to collagen degradation, blood-aqueous barrier breakdown, AC exudation, and progressive lens opacification.^8^^,^^23^ Consequently, by the time delayed SSOE is applied, pathological changes such as AC fibrin and cataract formation are established and cannot be reversed. Thus, it is plausible that SSOE given shortly after the burn reduces the actual penetration of alkali into the AC and the rapid cascade of intraocular inflammation, which once activated is difficult to reverse. It is also plausible that damaged lens epithelium from the initial burn inevitably triggers further lens opacification that cannot be rescued with delayed SSOE treatment. Additionally, we speculate that as the ocular tissue heals beyond the initial injury, permeation of oxygen from SSOE is limited to the ocular surface including the cornea but not the intraocular structure. These speculations warrant further investigation into how SSOE affects chemical permeation in the acute stage and intraocular hypoxia in the subacute stage.

Inflammation is central to the pathophysiology of corneal alkali burns and intertwined with tissue hypoxia, HIF activation, pathological angiogenesis, and VEGF signaling.^24^, ^25^, ^26^, ^27^, ^28^, ^29^, ^30^, ^31^, ^32^, ^33^, ^34^, ^35^ Hypoxia-inducible signaling pathways amplify inflammatory cascades, and inflammation itself perpetuates local oxygen imbalance. Recent work has highlighted this bidirectional relationship in other tissues, emphasizing the role of immune cell-derived metabolites and cytokines in sustaining hypoxic microenvironments.^20^ Our previous study on immediate 1-time–only SSOE treatment for alkali burn demonstrated significantly reduced tissue inflammation at the acute stage.^7^ Our current study shows that daily SSOE including delayed applications dampens leukocyte infiltration in the cornea. This suggests that SSOE, in addition to its potent antiangiogenic function, is anti-inflammatory. Whether its suppression of inflammation is HIF-dependent requires further investigation. Although immediate followed by daily SSOE application appears to have the best therapeutic efficacy in treating nearly all aspects of alkali burn, delayed SSOE treatment still potently inhibits corneal NV, fibrosis, and to a certain extent edema. Given that ophthalmic SSOE is packaged in a ready-to-use canister and directly applied to the eye without the need for additional manipulation,^7^ it may be a promising therapeutic where immediate care is unavailable, such as in remote or austere environments. These findings align with previous studies that demonstrate the benefits of prolonged oxygen therapies in mitigating tissue damage and fibrotic remodeling after ocular injury.^36^, ^37^, ^38^

While this study highlights the therapeutic potential and treatment window of SSOE, limitations include the use of a single injury model and relatively short follow-up periods. While the study focuses on hypoxia-induced HIF activation, it does not rule out or fully investigate other contributing pathways such as nuclear factor kappa B activation^39^^,^^40^ that might also play significant roles in the injury response. Moreover, we only focused on HIF-1α activation, which is the most expressed HIF variant; however, further studies are needed to delineate the HIF-2 expression in response to burn. Finally, the limbus was not directly exposed to alkali in the current model; thus, further research is needed to evaluate the impact of SSOE in preserving limbal stem cells. Studies to explore these areas and test SSOE in treating chemical eye injury in rabbits are ongoing.

In conclusion, this study highlights the critical role of persistent tissue hypoxia and HIF activation in ocular chemical burn and the effects of SSOE in targeting hypoxia-mediated pathways. Extended use of SSOE mitigates nearly all aspects of chemical burn-induced ocular damage including corneal NV, opacification, edema, intraocular inflammation, and cataract formation. Importantly, the efficacy observed with delayed treatment suggests that SSOE may be particularly beneficial in scenarios where immediate care is not possible.

## Data Availability

The other data generated during and analyzed during the current study are available upon request from the corresponding author.
